# Spectral Physics of Stable Cu(III) Produced by Oxidative Addition of an Alkyl Halide

**DOI:** 10.3390/ijms242115694

**Published:** 2023-10-28

**Authors:** En Cao, Mengtao Sun

**Affiliations:** 1 School of Mathematics and Physics, University of Science and Technology Beijing, Beijing 100083, China; e-cao@es.hokudai.ac.jp; 2 Research Institute for Electronic Science, Hokkaido University, Sapporo 001-0021, Japan

**Keywords:** spectral physics, stable Cu(III), produced, oxidative addition of, alkyl halide

## Abstract

In this paper, we theoretically investigated spectral physics on Cu(III) complexes formed by the oxidative addition of α-haloacetonitrile to ionic and neutral Cu(I) complexes, stimulated by recent experimental reports. Firstly, the electronic structures of reactants of α-haloacetonitrile and neutral Cu(I) and two kinds of products of Cu(III) complexes are visualized with the density of state (DOS) and orbital energy levels of HOMO and LUMO. The visually manifested static and dynamic polarizability as well as the first hyperpolarizability are employed to reveal the vibrational modes of the normal and resonance Raman spectra of two Cu(III) complexes. The nuclear magnetic resonance (NMR) spectra are not only used to identify the reactants and products but also to distinguish between two Cu(III) complexes. The charge difference density (CDD) reveals intramolecular charge transfer in electronic transitions in optical absorption spectra. The CDDs in fluorescence visually reveal electron–hole recombination. Our results promote a deeper understanding of the physical mechanism of stable Cu(III) produced by the oxidative addition of an alkyl halide.

## 1. Introduction

As a cheap and plentiful metal, Cu can occur in oxidation states from 0 to 4+, although +1 (cuprous) and +2 (cupric) oxidation states are the most common, while Cu with 0 and +4 oxidation states are very rare. High-valent organo-Cu(III) species were mostly considered the main intermediates in many aerobic oxidation reactions, enzyme-catalyzed oxidation reactions, as well as Cu-catalyzed cross-coupling reactions [1,2,3,4,5,6,7]. Reductive elimination of Cu(III) intermediates is frequently projected as an important step in various Cu-catalyzed or copper-mediated formation of C-heteroatom or C-C bonds. Organo-Cu(III) species are produced in several ways [7]: (1) two-electron oxidative addition of Cu(I) species; (2) oxidation of Cu(II) intermediate via a single-electron transfer pathway or via halide atom transfer [8,9]; (3) σ-bond metathesis through a four-centre intermediate; and (4) p-complexation of Cu(I) on ArX. In (1) and (2), the Cu species changes its oxidation state during the catalytic cycle, whereas in (3) and (4), the Cu species preserves the same oxidation state through the whole cycle. Usually, there are four types of well-defined Cu(III) complexes among the d^8^ Cu(III) complexes: (1) Cu(III) complexes without Cu-Carbon bond [10,11,12,13,14,15,16,17,18,19,20]; (2) organo-Cu(III) complexes supported by macrocyclic ligand and with Cu-C bond [21,22,23,24,25,26,27,28,29,30,31,32]; (3) fluoroalkylated organo-Cu(III) complexes [33,34,35,36,37,38,39,40,41,42,43,44]; and (4) organo-Cu(III) complexes containing a Cu(III)-C bond [45,46,47,48,49,50,51,52,53].

**Figure 1:**
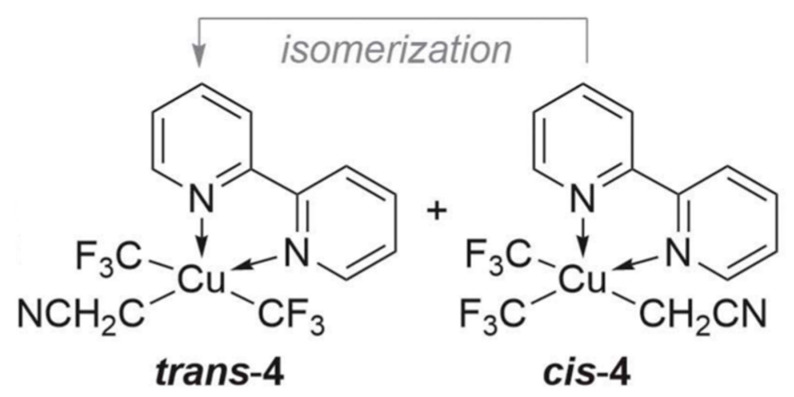
The trans-[(bpy)Cu(CF_3_)_2_(CH_2_CN)] (trans-4) and cis-[(bpy)Cu(CF_3_)_2_(CH_2_CN)] (cis-4). This figure was taken from ref. [57].

Contrary to lots of other high-valent transition metal complexes, Cu(III) complexes are usually thermally unstable and hard to isolate. (CF_3_)_2_CuS_2_CNE, as an organo-Cu(III) specie, was isolated and characterized (vide infra) in 1989 [54]. Before 2000, few well-defined Cu(III) complexes were obtained and well-characterized because of their low thermal stabilities [55]. In the early 2000s, with rapid-injection nuclear magnetic resonance (RI-NMR) spectroscopy, Cu(III) specie was demonstrated [22,23,24,56]. It is urgent to study the organo-Cu(III) complexes with thermal stability and their elementary bond-forming reductive elimination because these investigations can reveal the fundamentals of Cu catalysis and promote the discovery of new reactions. Recently, Shen et al. experimentally reported the oxidative addition of an alkyl halide to form a stable Cu(III) product (see Figure 1) [57], and this oxidative addition of a C(sp^3^) − X bond to Cu(I) can help promote more effectual Cu-catalyzed cross-coupling reactions of alkyl electrophiles.

In this paper, we theoretically investigate the spectral physics of stable Cu(III) produced by the oxidative addition of an alkyl halide, stimulated by the recent experimental report in ref. [57]. The electronic structures of reactants of α-haloacetonitrile and neutral Cu(I) and two kinds of products of Cu(III) complexes are analyzed with DOS and by measuring the orbital energy levels. The static and dynamic polarizability as well as the first hyperpolarizability reveal the vibrational modes of the normal and resonance Raman spectra of two Cu(III) complexes. The NMR spectra are not only used to identify the reactants and products but also to distinguish between two Cu(III) complexes. Absorption and fluorescence spectra and charge transfer in electronic transitions are manifested. Our results promote a deeper understanding of the physical and chemical properties of Cu(III) complexes.

## 2. Results and Discussion

**Figure 2:**
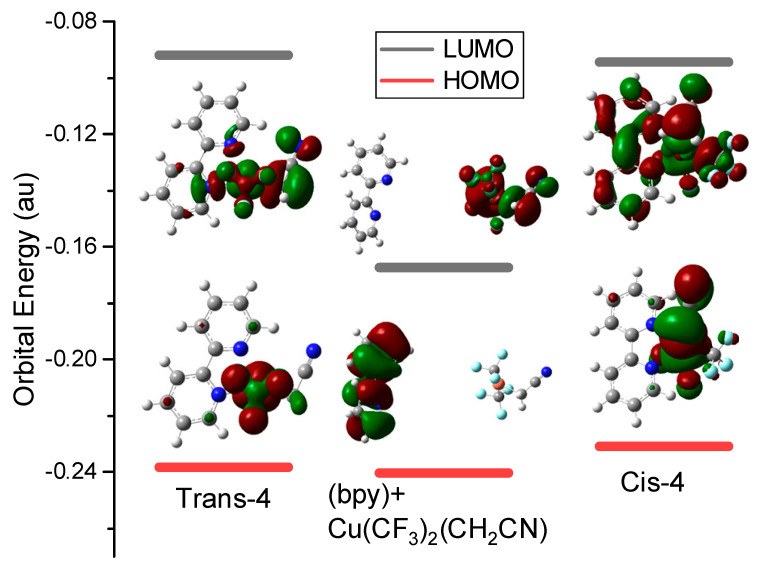
The orbital energy level and DOS of trans-4, cis-4, and (bpy) being far away from Cu(CF_3_)_2_(CH_2_CN) before complex.

Figure 2 shows the orbital energy level and DOS of trans-4, cis-4, and (bpy) being far away from Cu(CF_3_)_2_(CH_2_CN) before complex. Without interaction between (bpy) and Cu(CF_3_)_2_(CH_2_CN), the DOS of HOMO and LUMO are localized on (bpy) and Cu (CF_3_)_2_(CH_2_CN), respectively. When the complexes are formed, the DOS of HOMO are localized on the Cu CF_3_)_2_(CH_2_CN), and DOS are not only localized on Cu(CF_3_)_2_(CH_2_CN), but also on the (bpy), which directly reflects the interaction between (bpy) and Cu(CF_3_)_2_(CH_2_CN), especially for cis-4, and this interaction results in an increase in the orbital energy level of LUMO for the complexes.

**Figure 3:**
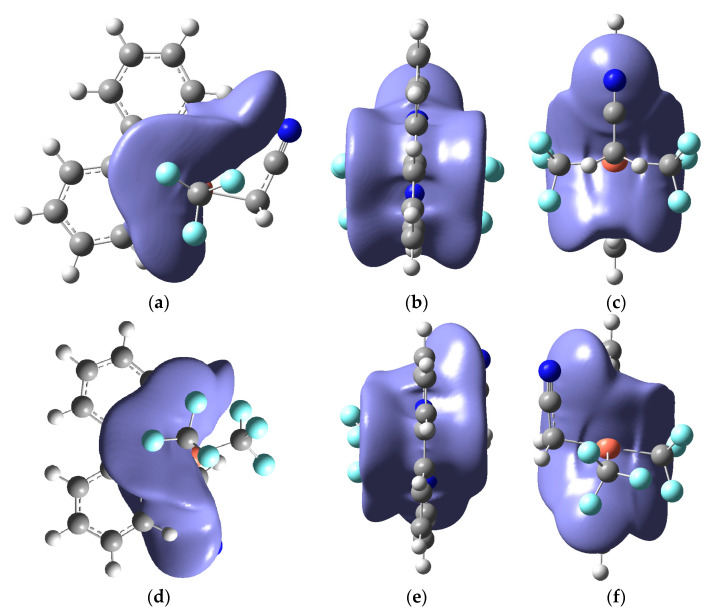
Interaction between (bpy) and Cu(CF_3_)_2_(CH_2_CN) for trans-4 and cis-4 with top and side views, (**a**–**c**) for trans-4, and (**d**–**f**) for cis-4.

Figure 3 demonstrates the noncovalent interactions (NCI) plotetween (bpy) and Cu(CF_3_)_2_(CH_2_CN) for trans-4 and cis-4, respectively, with top and side views. It was found that there is a strong attraction between (bpy) and Cu(CF_3_)_2_(CH_2_CN), which is the key factor in forming stable complexes. The visual interaction also clearly demonstrates the influence of the (CH_2_CN) position on the difference in interaction between trans-4 and cis-4.

**Figure 4:**
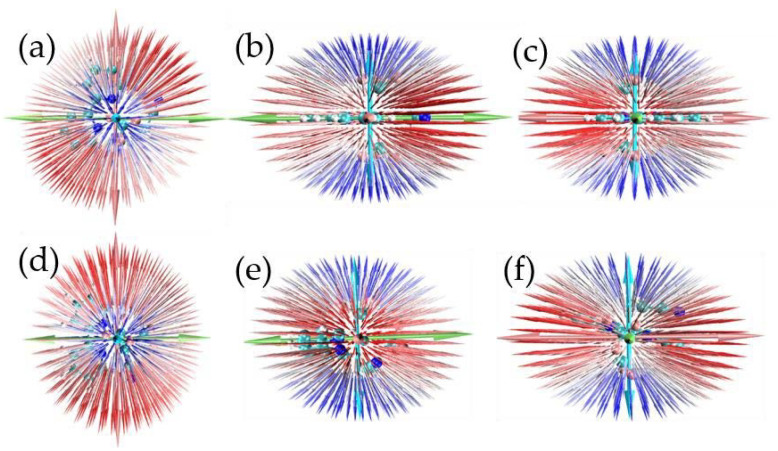
The permanent polarizability of trans-4 and cis-4 with top and side views: (**a**–**c**) top and side views of trans-4; (**d**–**f**) top and side views of cis-4.

**Figure 5:**
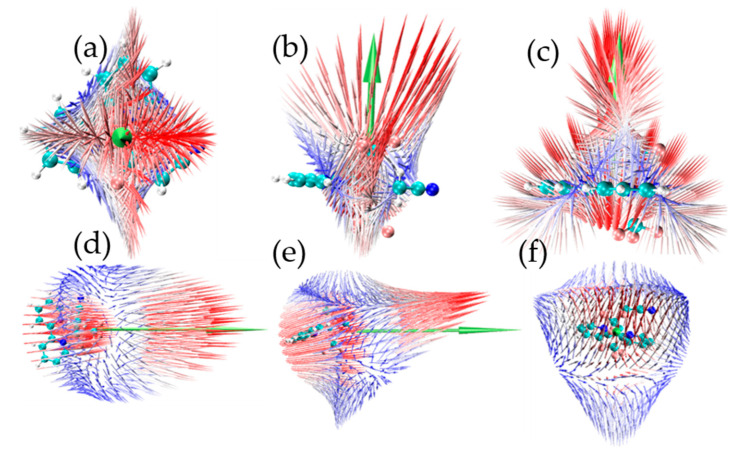
The permanent polarizability of trans-4 and cis-4 with top and side views: (**a**–**c**) top and side views of trans-4; (**d**–**f**) top and side views of cis-4.

Trans-4 is more stable than cis-4 since the ground energy of trans-4 is lower (0.1864 eV) than that of cis-4. The ground state permanent dipole moment energy refers to the permanent dipole moment possessed by a molecule in the ground state. It is usually represented by μ, which can be used to measure the interaction ability between molecules and external electric fields. Polarization rate is a physical quantity that describes the degree of polarization of a molecule or material under the effect of an external electric field. It describes the ability to react to external electric fields. The symbol for polarizability is usually α; the larger its value, the easier the material is to polarize under external electric fields, and vice versa. Hyperpolarizability is a physical quantity that describes the response of a material to an external electric field and can reflect its nonlinear optical properties. The symbol for hyperpolarizability is usually β. The data on ground permanent dipole moment, polarizability, and the first hyperpolarizability are listed in Table 1 and Table 2. It is found that there are similar ground dipole moments and polarizability between trans-4 and cis-4 (see Table 1), and the visualization of polarizability supports the above conclusion in Figure 4, but there is a large difference in the first hyperpolarizability in Table 2 and Figure 5. In Figure 4 and Figure 5, the blue, white, and red colors represent the polarizability from small to large. The arrows on the spherical surface in the figure represent the change in the dipole moment of the molecule when the center of the molecule applies an external electric field of the same strength in all directions. The green, pink, and light blue long arrows represent the general trend of the polarizability of the system along three different orientations.

**Figure 6:**
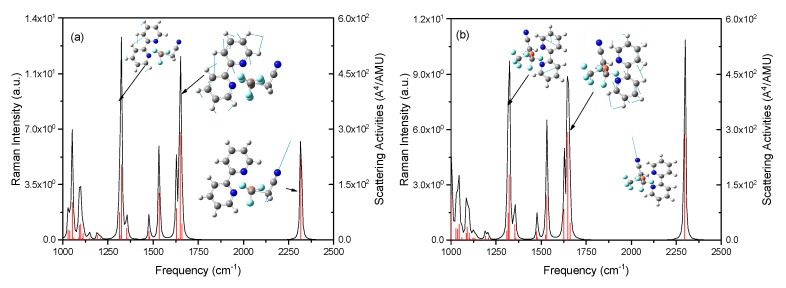
Normal Raman spectra of trans-4 and cis-4: (**a**) rans-4; and (**b**) cis-4.

Figure 6 shows the normal Raman spectra of trans-4 and cis-4. It is found that their profiles are almost the same because of their similar permanent polyaxiality, which can be seen in Figure 4, since Raman intensity (I) is estimated with *I* = α^2^*E*^4^, where α is permanent polarizability. The green, pink, and light blue long arrows in Figure 4 represent the general trend of the polarizability of the system along three different orientations, which determines the molecular vibrational modes along the direction of the vector arrow’s direction of polarizability. The insert in Figure 6 demonstrates the vibrational modes of trans-4 and cis-4, respectively. Due to the different orientations of (CH_2_CN), their Raman intensities are significantly different.

**Figure 7:**
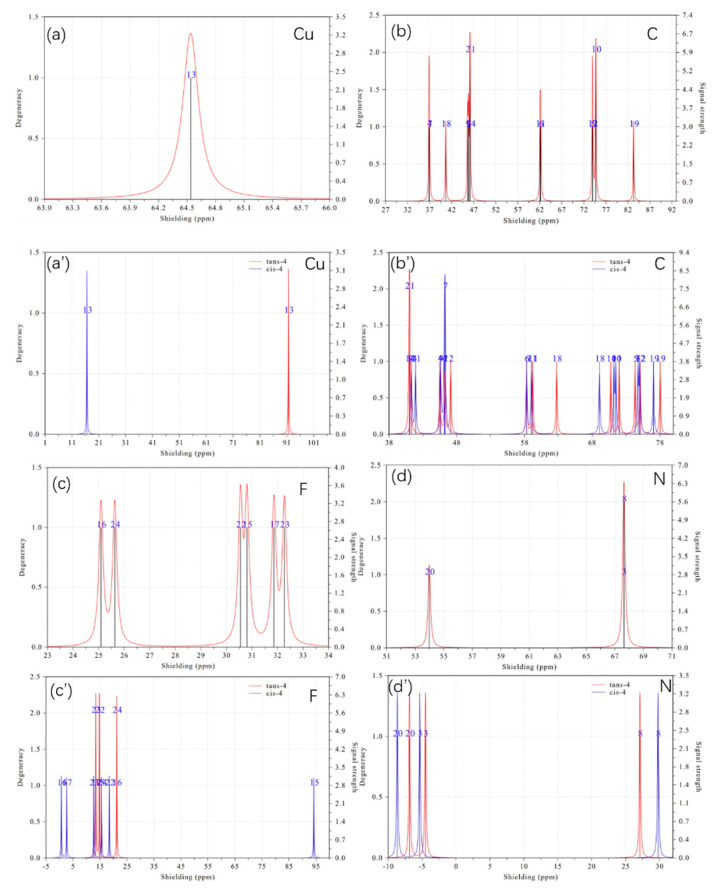

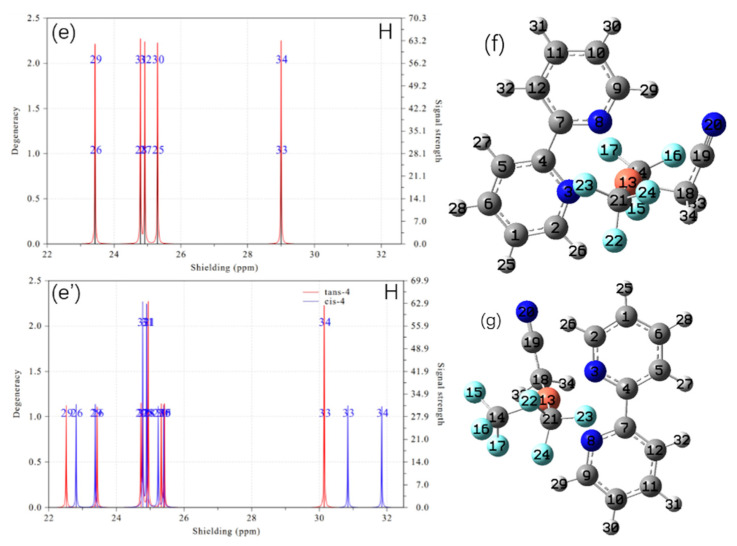
NMR spectra of Cu, C, F, N, and H atoms before and after they formed trans-4 and cis-4, which can also be used to distinguish trans-4 from cis-4. The parts (**a**–**e**) are before complex; (**a’**–**e’**) trans-4 and cis-4; and (**f**,**g**) trans-4 and cis-4 with labels, respectively.

NMR spectroscopy is a technique that provides information about the quantities and types of atoms of a particular element in a molecule. Figure 7a–e are the NMR spectra of Cu, C, F, N, and H atoms of (bpy) being far away from Cu(CF_3_)_2_(CH_2_CN) before complex, respectively. Figure 7a’–e’ are the NMR spectra of Cu, C, F, N, and H atoms of trans-4 and cis-4, respectively, which can distinguish trans-4 from cis-4. Figure 7f,g are the trans-4 and cis-4 with labels, respectively. By comparing the NMR spectra of each atom before and after reactions, we can see that the NMR spectra of Cu, F, N, and H can well characterize the formation of trans-4 and cis-4 because of the large shielding. For example, the NMR of the Cu spectrum demonstrates that shielding is 63.46 ppm, while the shielding of trans-4 and cis-4 is 16.5 ppm and 91.1 ppm; thus, NMR can not only identify whether the C(III) complex formed but can also distinguish trans-4 from cis-4. Also, NMR spectra of F demonstrate that there is a lot of shielding, especially the shielding of atom #15, which is 94 ppm for cis-4, as can be seen in Figure 7c’, while the shielding of atom #15 of Cu(CF_3_)_2_(CH_2_CN) is about 30.9 ppm. The shielding of degenerated N#3 and N#8 of (bpy) is 66.8 ppm, which can be seen in Figure 7d, which are separated to −5 ppm and 28 ppm, respectively. The shielding of N#20 of CH_2_CN is 54 ppm in Figure 7d, which is separated to −7 ppm and −9 ppm, respectively, for trans-4 and cis-4. By comparing Figure 7e,e’, one can examine whether the trans-4 and cis-4 are formed. Furthermore, the NMR spectra of the H atom for trans-4 and cis-4 show a great difference, which can also be used to distinguish between trans-4 and cis-4 in Figure 7e’.

**Figure 8:**
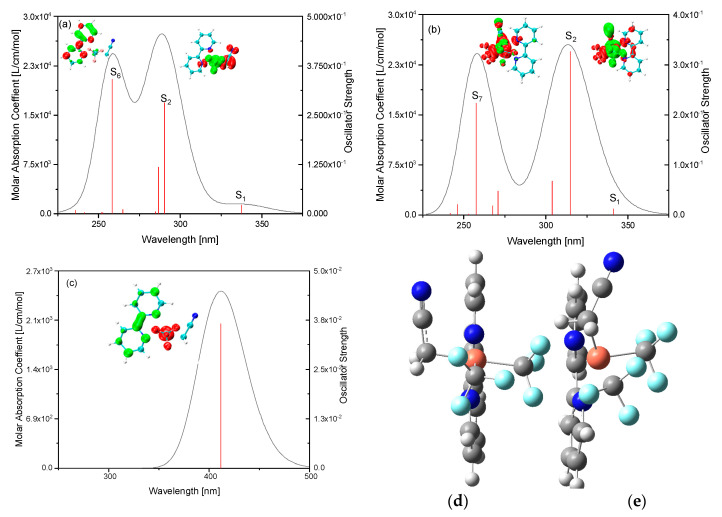
Absorption and fluorescence spectra of trans-4 and cis-4: (**a**,**b**) absorption spectra of trans-4 and cis-4, where green and red stand for hole and electron, respectively; and (**c**) fluorescence of absorption spectra of trans-4, and geometries before (**d**) and after (**e**) excited state optimization of cis-4.

Electronic transitions corresponding to optical absorption and fluorescence spectra can be seen in Figure 8, where the inserts are the CDDs. Figure 8a,b shows the absorption spectra of trans-4 and cis-4, respectively. CDDs demonstrate electron transfer from Cu (CF_3_)_2_ to (bpy) and (CH_2_CN) for trans-4 at the absorption peak around 300 nm, while for cis-4, they demonstrate electron transfer from Cu(CF_3_)(CH_2_CN) to (CF_3_) and (bpy). While for the peak around 250 nm, they both exhibit intramolecular charge transfers, but within (bpy) and Cu(CF_3_)_2_(CH_2_CN), respectively. Figure 8c manifests that the fluorescence is around 410 nm and electron transfer is from (bpy) to Cu(CF_3_)_2_ in electron–hole recombination in fluorescence. The excited geometry optimization of cis-4 from the structure in Figure 8d was performed, but it did not converge and stopped at the structure in Figure 8e, which demonstrates that the structure of cis-4 was broken, where (CH_2_CN) moved to one side of (bpy). The intramolecular charge transfer between the organic component and Cu(III) can further promote stability by optical excitation, which reflects the stability of the sample explored in light atmospheric environments.

**Figure 9:**
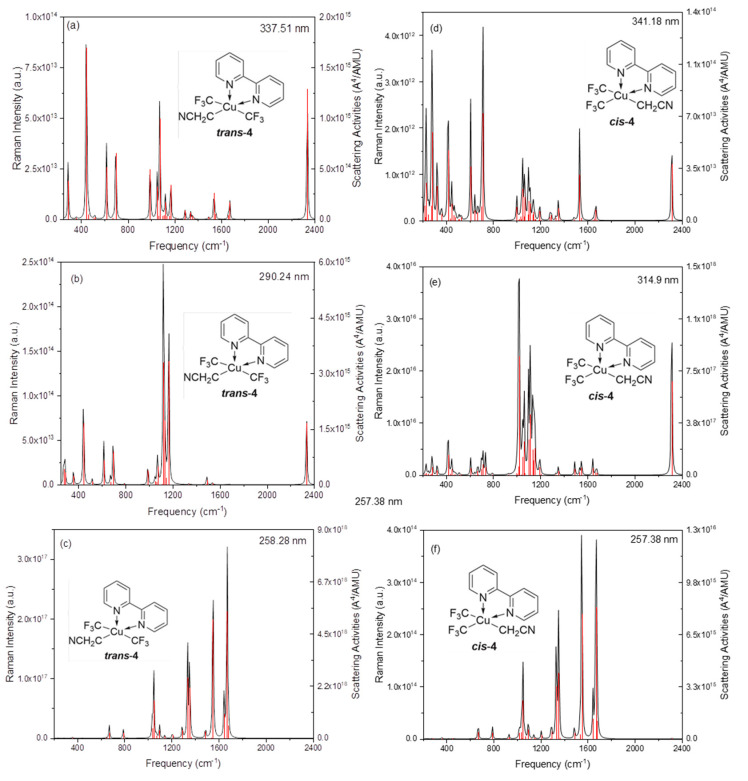
Resonance Raman spectra: (**a**–**c**) resonance Raman spectra of trans-4; and (**d**–**f**) resonance Raman spectra of cis-4.

**Figure 10:**
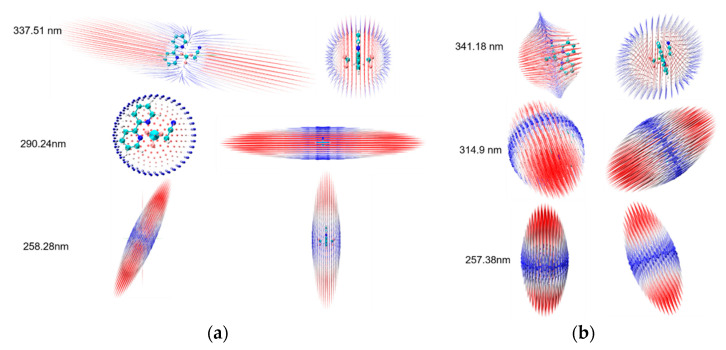
Dynamic polarizability: (**a**) dynamic polarizability of trans-4; and (**b**) dynamic polarizability of cis-4.

It can be seen that the normal Raman spectra of trans-4 and cis-4 in Figure 6 are similar and have weak Raman intensities due to the similar static permanent polarizability at the ground state, as can also be seen in Figure 7. To significantly increase the Raman intensities, as can be seen in Figure 6, the resonance Raman spectra of trans-4 and cis-4 are calculated; see Figure 9. Figure 9a–c shows the resonance Raman spectra of trans-4, excited by different resonant electronic state excitations. Dynamic polarization rate is one of the types of polarization rates that is mainly used to describe the polarization response of molecules or materials under high-frequency electric fields. It is found that resonance Raman spectra in Figure 9a–c are significantly selectively enhanced by charge transfer enhancement, resulting from the different dynamic polarizability at different incident lights; see the visualization with top and side views in Figure 10a. There are similar results for the resonance Raman spectra of cis-4 shown in Figure 9d–f, which are significantly enhanced by dynamic polarizability on different resonant electronic transitions in Figure 10b.

## 3. Materials and Methods

All calculations were performed with Gaussian 16 software [58] based on density functional theory (DFT) [59]. The geometries of trans-4 and cis-4 were optimized at ground state. B3LYP functional [60] and 6-31G(d) basis sets were used for C, N, F, and H, and the LANL2DZ basis set was used for Cu. With the optimized geometries, the electronic structures, the noncovalent interactions (NCI) [61], (hyper) polarizability, normal Raman spectra, and NMR spectra were calculated at the same level of the theory. Electronic transitions in absorption and fluorescence and resonance Raman spectra were calculated with time-dependent DFT (TD-DFT) [62], CAM-B3LYP functional [63], and 6-31G(d) basis sets for C, N, F, and H, and the LANL2DZ basis set for Cu. The geometries of the S_1_ excited state for trans-4 and cis-4 were optimized, with TD-DFT, B3LYP functional, and 6-31G(d) basis sets being used for C, N, F, and H, and the LANL2DZ basis set being used for Cu. With the optimized geometries of the S_1_ excited state, the fluorescence of trans-4 and cis-4 was calculated with TD-DFT and CAM-B3LYP functional, with the 6-31G(d) basis set being used for C, N, F, and H, and the LANL2DZ basis set being used for Cu. The visualizations of polarizability were performed according to refs. [64,65,66].

## 4. Conclusions

Stable Cu(III) produced by the oxidative addition of an alkyl halide was theoretically investigated using the spectral analysis method. NMR spectra can not only reveal the dynamic processes of forming trans-4 and cis-4 but also distinguish trans-4 from cis-4. Resonance Raman spectra are selectively and significantly enhanced by chemical enhancement, which can also distinguish trans-4 from cis-4. CDDs of electronic transitions reveal the electron–hole separation in optical absorption processes and can manifest the electron–hole recombination in fluorescence. Our theoretical analysis provides an understanding of the physical mechanism of stable Cu(III) produced by the oxidative addition of an alkyl halide.

## Figures and Tables

**Table 1:** The permanent dipole moment (Debye) and polarizability (au) of trans-4 and cis-4 at ground state, where data are the total values.

	μ (x)	μ (y)	μ (z)	α(xx)	α(yy)	α(zz)
trans-4	−9.0	0.7	0.0	237	231	128
cis-4	10.2	−2.7	−1.4	215	253	139

**Table 2:** The permanent first hyperpolarizability (au) of trans-4 and cis-4 at ground state.

	β(xxx)	β(xxy)	β(xxz)	β(xyy)	β(xzz)	β(yyy)	β(yyz)	β(yzz)	β(zzz)
trans-4	340	−26	0	102	−215	133	0	−48	0
cis-4	−610	−15	51	−67	110	−41	−70	9	184

## Data Availability

Data are available on request from authors.
